# Sleep Loss and Substance Use Disorders: An Issue from Adolescents to Adults

**DOI:** 10.3390/bs15020220

**Published:** 2025-02-15

**Authors:** Ana Clementina Equihua-Benítez, Rodolfo Espinoza-Abad, Fabio García-García

**Affiliations:** 1 Biology Sleep Laboratory, Biomedicine Department, Health Sciences Institute, Veracruzana University, Xalapa 91190, Ver, Mexico; aequihua@uv.mx; 2 Graduate Program in Health Sciences, Health Sciences Institute, Veracruzana University, Xalapa 91190, Ver, Mexico; rodespinoza@uv.mx

**Keywords:** substance use disorders, insomnia, orexin, hypocretin, addiction

## Abstract

Unsatisfactory sleep is a worldwide concern, as evidenced by the high prevalence of insomnia symptoms and diagnosis in the general population, and an issue that has also risen among adolescents. These circumstances are a cause of worry due to, among other factors, the observed bidirectional association of sleep disturbances and the risk of substance use disorder development. In this regard, across the globe, several reports indicate that substance consumption is at an all-time high, with alcohol, nicotine, and cannabis leading the charts. Additionally, the age of onset has dropped, with reports suggesting that first contact is usually during adolescence. Although the nature of the link between poor sleep and substance use disorder development is still not fully understood, it is possible that an overactive orexinergic system could play a role, as it has been observed that treatment with orexinergic antagonists improves insomnia symptoms and that postmortem studies show an increase in orexin immunoreactive neurons in sections obtained from habitual opioid consumers. We further argue that it is during adolescence that this maladaptive loop can be established, priming for the development of substance use disorders.

## 1. Substance Use: A Worldwide Health Concern

Drug use is a global concern heavily impacting individuals and societies. On a personal scale, indiscriminate consumption is known to have deleterious health effects that range from increased risk of disease and accidents, diminished quality of life, and, in some extreme cases, death. Simultaneously, societies are negatively impacted when health services become strained, productivity decreases, and bystanders are affected, e.g., through secondhand smoke exposure, traffic incidents, and neonatal abstinence syndrome, among others (Levy et al., 2017).

The extent of the issue is significant, as evidenced by reports published by the United Nations (UN) and the World Health Organization (WHO). On this subject, the UN mentions in their 2023 World Drug Report, a document following the use of drugs such as cannabis, opioids, and stimulants, that in 2021, around 296 million people between the ages of 15 and 64 declared having consumed one or more of these drugs in the past 12 months, a number that is a 23% increase regarding the number reported by the same organization a decade before (UNODC, 2023). Meanwhile, in their report, the WHO states that consumers of alcohol and nicotine range in the billions (2.5 and 1.25 billion, respectively) (World Health Organization, 2024). Furthermore, alarming numbers show that every year across the globe, millions of premature deaths are linked to substance misuse, a trend that has been steadily increasing year after year. In this regard, the WHO reports that for the year 2019, alcohol consumption caused around 2.9 million deaths worldwide, while psychoactive drugs accounted for nearly 600,000 (World Health Organization, 2024).

Additional concerns arise when considering that substance use among youths is widespread and that, while varying according to drug type, the age of first contact usually happens somewhere during adolescence, with some cases occurring even before the age of 11 (A. Strashny, 2014). These numbers are particularly worrying as a low age of substance use onset is strongly related to worse mental, behavioral, and health outcomes, along with an increased risk of developing a substance use disorder (SUD) (Poudel & Gautam, 2017).

In consequence, as global concern for substance use grows, research interest aimed at understanding how SUDs develop and perpetuate has also increased in hopes of improving treatment options and strengthening prevention strategies. In this regard, as we will see in the following sections, there is ample evidence that SUDs are often accompanied by troubled sleep and that adolescence is a particularly vulnerable time point for sleep issues to arise.

## 2. SUDs and Sleep Disorders

SUDs are a group of mental health conditions where there is an underlying pattern of substance misuse that negatively impacts an individual’s health, causing impaired functionality and/or distress (Volkow & Blanco, 2023). In this context, evidence indicates that substance use and sleep issues are frequently found together. For example, Roncero et al. studied sleep complaints among patients admitted for detoxification and reported that around 68% of them manifested having a sleep disorder, with the majority complaining of insomnia or hypersomnolence. Furthermore, the authors suggested that the degree of sleep disturbances was related to the gravity of the SUD, as poly-drug users and patients with previous hospitalizations reported worse insomnia than single-drug users and first-timers, respectively (Roncero et al., 2012).

In many cases, substance use begins as self-medication to treat underlying sleep issues that, in turn, become exacerbated by the substance use, causing the individual to continue consuming or to relapse during withdrawal as sleep disturbances escalate and worsen the negative effects of different addictive substances (Gordon, 2019). Such is the case of alcohol consumption, which is a common recourse for inducing sleep among non-alcoholic people with insomnia and can lead to alcohol use disorder (AUD). In this regard, alcohol is commonly used as a sleep aid for its soporific effects, as it can help with sleep initiation by reducing sleep onset latency and improving slow-wave sleep (Brower, 2001). Nonetheless, tolerance to alcohol quickly builds, requiring higher doses to achieve the desired sleep-promoting effect (Roehrs & Roth, 2018). With dose escalation, sleep quality is negatively affected, as sleep fragmentation and arousals increase during the second half of the night, and breathing issues such as snoring and obstructive sleep apnea worsen (Chakravorty et al., 2016). A similar situation occurs among shift workers, a cohort that has been demonstrated to sleep less than day workers and to suffer from difficulties in initiating and maintaining sleep and to also frequently use alcohol as a sleep aid (Richter et al., 2021).

While most available research focuses on alcohol consumption, evidence shows that many addictive substances impair sleep and that, in particular, their use tends to diminish sleep efficiency and reduce total sleep time in current users and those going through withdrawal (one week or more), as described by Gordon et al., for the case of cocaine, nicotine, cannabis, and opioids (Gordon, 2019). Furthermore, among patients with different SUDs, persistent sleep issues have been identified as a risk factor for relapses among the population attempting recovery, where insomnia symptoms encompass the most frequent complaints (Brower, 2003; Erga et al., 2022; Garcia & Salloum, 2015). Recent evidence continues to support this notion, as a study carried out on patients recovering from opioid use disorder found that around 60% of participants had clinically significant insomnia, which was recognized by many of them as an obstacle in their recovery and coinciding with worse mental health outcomes in domains such as depression, stress, and anxiety (White et al., 2024). These observations have led many authors to suggest that there is a bidirectional link between both phenomena, where sleep issues increase the risk of developing an SUD and substance use alters sleep, facilitating the development of sleep disorders (Cañellas & De Lecea, 2012; Navarro-Martínez et al., 2020).

## 3. Sleep Loss During Adolescence and the Risk of Developing an SUD

Sleep deprivation in adults is associated with an increase in the likelihood of substance use, and at least some of these reported sleep issues may commence early in life as, in recent years, it has been reported that adolescents tend to sleep less than the evidence-based recommended amount. According to the WHO, adolescence is the period of life that spans from ages 10 to 19, where rapid physiological, physical, and cognitive growth occurs. For this life stage, the American Academy of Sleep Medicine states that the recommended daily amount of sleep is around 9–12 h for children aged 6–12 and at least 8–10 h for older teenagers between the ages of 13 and 18 (Paruthi et al., 2016). While these times are optimal, many studies have found that adolescents are not sleeping enough. For example, after analyzing data from several surveys to determine the prevalence of short sleep durations in children, the Centers for Disease Control and Prevention in the US found that almost 60% of middle schoolers (ages 11–13) and 70% of high schoolers (ages 14–18) sleep less than the recommended amount (Wheaton et al., 2018). This trend has also been observed in studies carried out in other countries (Galan-Lopez et al., 2021; Gariepy et al., 2020; Maduabuchi et al., 2014).

The lack of sleep among adolescents is a concerning issue, as studies carried out in recent years have confirmed that among this population, insufficient sleep also correlates with an increased likelihood of substance use. In this regard, studies show that trouble sleeping that continues from childhood into adolescence can predict illicit drug use among young adults (Wong et al., 2009). This has been argued, for example, by Mike et al., who carried out a longitudinal study that compared results obtained from the Child Sleep Questionnaire at age 11 with results from a follow-up interview regarding lifetime cannabis and alcohol consumption at ages 20 and 22, finding that reduced sleep duration during adolescence was associated with earlier first use, intoxication, and the repeated use of both alcohol and cannabis (Mike et al., 2016). Other longitudinal studies conducted among high-school and secondary-school adolescents have also shown that those who experience shorter sleep durations and poorer sleep quality are more likely to use substances such as alcohol and engage in binge drinking episodes within the following year (Dokkedal-Silva et al., 2020; Hasler et al., 2022; Haynie et al., 2018; Leger et al., 2022; Liu et al., 2023; Miller et al., 2017; Nguyen-Louie et al., 2018; Zahran & Janssen, 2023). These results appear to be particularly true for substances such as alcohol, tobacco, methamphetamines, and marijuana (Dokkedal-Silva et al., 2021; Leger et al., 2022; Roehrs et al., 2021; Wheeler et al., 2021).

Therefore, studies show that in both adults and adolescents, impaired sleep increases the risk of substance use. It also describes that difficulty falling asleep and reduced sleep quality are sleep complaints often found along with problematic substance use. Dissatisfaction with the quantity or quality of sleep is a symptom of insomnia, a sleep disorder that affects roughly one-third of the adult population (Ohayon, 2011; Zweig et al., 2024). In the US alone, a survey carried out by the American Academy of Sleep Medicine found that around 12% of American adults have been diagnosed with chronic insomnia, with individuals aged between 25 and 34 being the most affected age group (American Academy of Sleep Medicine, 2024).

As per the third edition of the International Classification of Sleep Disorders (ICSD-3), insomnia is a disorder characterized by disturbed sleep, where difficulty with sleep initiation or maintenance despite adequate opportunity to sleep causes significant daytime impairment (Deak & Winkelman, 2012). While in adults discomfort with sleep is usually expressed as taking too long to fall asleep and waking often or too early, in children and adolescents, sleep issues are more often reported by caregivers and observed as bedtime resistance or difficulty sleeping without the aid of the caregiver; likewise, the caregivers more often notice daytime impairment in the child.

Although insomnia in adolescents is difficult to evaluate due to the many challenges faced during this life stage that interfere with the maintenance of healthy sleep patterns, reports demonstrate that insomnia is also common in this age group, with evidence showing a prevalence rate of 18.5% for older adolescents (16–18 y) (de Zambotti et al., 2018).

## 4. Adolescence: A Perfect Storm for Sleep Issues

The development of insomnia is often explained using the 3P model proposed by Spielman et al.; this model considers that there are predisposing, precipitating, and perpetuating factors that drive apparently normal sleep into insomnia (Spielman et al., 1987). Predisposing factors include a wide range of traits, from genetic disposition to neurobiological mechanisms and personality attributes that make someone more vulnerable to insomnia. Precipitating events are regarded as the occurrence of a stressor that causes an initial insomnia episode that is considered acute. As time passes, the precipitating event usually resolves, but the insomnia continues due to perpetuating factors, such as maladaptive coping behaviors and learned negative associations that maintain high levels of stress and feed the insomnia cycle (Lancee et al., 2017; Riemann et al., 2023).

For adolescents, Crowley et al. have explained sleep problems associated with this age by using the ’perfect storm’ model, where psychosocial and societal pressures, in combination with the maturation process characteristic of adolescence, result in short and ill-timed sleep (Crowley et al., 2018; Hatori et al., 2017; Lund et al., 2021). Maturation influences the timing of circadian rhythms, as it has been noted that during adolescence, beginning with puberty, sleep and circadian rhythms gradually shift to later times, reaching their latest point around the age of 20 (Hagenauer et al., 2009). This shift is partially influenced by neurobiological mechanisms that alter the endogenous circadian system, leading to a delayed sleep phase and a slower accumulation of sleep pressure, facilitating later sleep times among adolescents (Crowley et al., 2014). After this peak, circadian rhythms begin to shift to earlier times again, continuing this trend throughout the rest of life (Gariepy et al., 2020; Gradisar et al., 2011). Although biological factors encompass shifts in circadian rhythms and alterations in homeostatic sleep propensity during adolescence, external factors are also involved. In particular, the unrestricted use of electronic devices by adolescents is a significant contributor to sleep issues, as it increases exposure to blue light emissions, a light frequency that efficiently inhibits melatonin secretion, causing delayed sleep onset and reduced sleep efficiency, factors that can intensify the natural inclinations towards delayed sleep and later circadian timing (Chang et al., 2015; Figueiro & Overington, 2016; Pérez-Chada et al., 2023; Perrault et al., 2019; Santiago et al., 2022; Silvani et al., 2022).

In addition, the decrease in sleep duration is partly caused by a divergence between the natural tendency for later sleep and circadian timing and by the imposed early start times of school, especially in middle and high school (Goldin et al., 2020; Meyer et al., 2022; Roenneberg, 2023). This mismatch, known as circadian misalignment or social jet lag, not only leads to insufficient sleep but also contributes to challenges falling asleep on school nights, daytime drowsiness during school days, and significant variations in sleep timing and duration between weekdays and weekends (Crowley et al., 2014; Gariepy et al., 2020; Vazsonyi et al., 2021). These variations often appear as later sleep timing and reduced sleep duration, particularly among individuals with naturally later circadian rhythms (Crowley et al., 2014; Gariepy et al., 2020).

In other words, adolescents are at a high risk of developing chronic insomnia due to the challenges related to this life stage as described by the perfect storm model (de Zambotti et al., 2018), where predisposing (maturation process), precipitating (school stress, electronic device use), and perpetuating (early school start) factors are maintained for a long time, which in turn conveys the added risk of developing an SUD.

During this stage, structural, functional, and neurochemical modifications occur in the brain, significantly influencing behavior, decision-making, emotional regulation, and mental health (Fuhrmann et al., 2015; Luna et al., 2015). One of the most notable changes is the maturation of the prefrontal cortex, the last brain region to reach full development. This area plays a crucial role in executive functions, including decision-making, planning, impulse control, and working memory skills that become fully established towards the end of this stage (Blakemore & Robbins, 2012; van Duijvenvoorde et al., 2016).

In contrast, the limbic system, which includes structures such as the amygdala and the nucleus accumbens, matures at a faster rate than the prefrontal cortex. This asynchrony leads to heightened emotional reactivity and increased sensitivity to the brain’s reward system, promoting the pursuit of novel and risky experiences, hallmark behaviors of adolescence (Casey et al., 2008; Steinberg, 2008; Tottenham & Galván, 2016).

Sleep deprivation also disrupts the functional connectivity between the prefrontal cortex and the amygdala, both essential for emotional regulation. Additionally, it enhances nucleus accumbens activity, increasing sensitivity to pleasurable stimuli and reinforcing the tendency to seek immediate rewards (Mullin et al., 2013). This phenomenon, linked to increased dopamine release during adolescence, contributes to heightened impulsivity and greater vulnerability to risk-taking behaviors (Parr et al., 2022; Toth, 2022).

In sleep-deprived adolescents, there is a marked preference for high-risk rewards, such as substance use, reckless driving, or gambling, due to a diminished ability to anticipate negative consequences (Hisler et al., 2023). Furthermore, dopamine follows circadian rhythms disrupted by sleep deprivation, leading to dysregulated release and negatively impacting the homeostasis of the reward system (Wu et al., 2024).

## 5. Excessive Orexinergic Tone in Insomnia and Addiction

Since their first description at the end of the 20th century (de Lecea et al., 1998; Sakurai et al., 1998), the orexinergic system has been heavily implicated in the maintenance of vigilance states through the stabilization of the so-called flip-flop switch that regulates transitions from wake to sleep (Saper et al., 2001) and by avoiding inappropriate intrusions of sleep phenomena into wakefulness such as those observed in the course of narcolepsy, a sleep disorder characterized by disorganized sleep that has been tied to orexinergic deficiency in humans (Mignot et al., 2002; Thannickal et al., 2000).

It has been suggested that the predisposing factors among insomnia-prone individuals are related to a relative increase in cognitive, emotional, physiological, and cortical arousal maintained throughout the day, a state termed hyperarousal (Dressle & Riemann, 2023). Evidence of this hyperarousal has been observed in adults through the measurement of different parameters that suggest that insomnia patients tend to have elevated thresholds across many markers such as body temperature, blood pressure, heart rate, cortisol and adrenaline levels, skin resistance, increased global brain metabolism, and cortical activation (Bonnet & Arand, 2010; Dai et al., 2024; Dressle et al., 2022; Ma et al., 2024; Nofzinger et al., 2004). In addition, standardized sleep studies, such as the multiple sleep latency test (MSLT), have repeatedly demonstrated that insomnia patients have higher latencies to sleep (Shi et al., 2022; Stepanski et al., 1988), suggesting less sleepiness in these individuals, a counterintuitive finding considering the disturbed sleep insomnia patients present with. For the adolescent population, some evidence of this hyperarousal exists; for example, it has been shown that adolescents (16.6 ± 2 y) with insomnia have increased beta activity (15–35 Hz) during sleep onset and sleep (Fernandez-Mendoza et al., 2016), in addition to an increase in cortisol levels measured in saliva (Zhang et al., 2014).

While the mechanisms driving hyperarousal in insomnia are not yet fully understood, overactive orexinergic neurotransmission may play a role, as this excitatory system extensively innervates the central nervous system (Peyron et al., 1998) and has been associated with the promotion and maintenance of wakefulness across the sleep–wake cycle (Saper et al., 2001). Although orexins are recognized for their role in allowing wake and sleep to occur at the appropriate times, evidence suggests that heightened orexinergic activity can sustain vigilance states even as sleep pressure builds. This has been observed in many animal models. For example, in a zebrafish insomnia model, the genetically induced overexpression of orexin peptides resulted in an increase in locomotion and restlessness (Prober et al., 2006). In an O/E3-null mouse model of narcolepsy, for a mouse with a significant reduction in orexinergic somas and an increase in time spent in sleep, the intracerebroventricular infusion of orexin A peptide sharply suppressed sleep (De la Herrán-Arita et al., 2011). Furthermore, in the squirrel monkey, a primate with a sleep–wake cycle similar to that of humans, the measurement of orexin peptide levels in cerebrospinal fluid showed that in undisturbed animals, orexin levels varied across the day, with their lowest level being recorded around the wake onset time and a gradual increase occurring throughout the afternoon, with the levels stabilizing in the evening before dropping progressively during sleep. Nonetheless, in these animals, an artificial extension of the wake period correlated with the sustained production of orexin peptides to levels similar to those observed at their peak, varying from those registered for the time point under normal conditions, suggesting a role for orexins in helping sustain the vigilance state beyond regular sleep-onset times (Zeitzer et al., 2003). Together, this evidence indicates that the activity of the orexinergic system favors vigilance even as the need for sleep accumulates, an argument that has led some authors to propose that orexinergic hyperactivity could play a role in the hyperarousal that functions as a predisposing factor in insomnia (Palagini et al., 2023).

While more research carried out on humans is warranted, and information from the adolescent population is lacking, some reports support the relationship between increased orexin activity and insomnia, with results that are in tune with those obtained from research conducted on animal models. For example, it has been documented that insomnia patients possess elevated concentrations of circulating orexin A peptides compared to normal sleepers and that the concentrations found relate to the severity of the reported insomnia (Tang et al., 2017). In addition, many studies have found that orexin stimulation influences the autonomic system; hence, an increased orexinergic tone could explain some of the abnormal autonomic markers found in insomnia patients. Such is the case of cardiac function and temperature abnormalities, as orexin activity has been linked to increased blood pressure and heart rate (Carrive, 2017), while those with narcolepsy tend to display lower body temperatures (Wang et al., 2023). Moreover, the inhibition of the orexinergic system has become a successful therapeutic target that has been proven to be safe and effective in alleviating insomnia symptoms (Khazaie et al., 2022), with three Food and Drug Administration-approved orexinergic antagonists, suvorexant, Lemborexant, and daridorexant (Shaha, 2023), already in the market and a fourth compound, vornorexant, undergoing clinical trials (Konno et al., 2024), indirectly suggesting that orexinergic activity is dysregulated among those with insomnia.

More recently, orexins have also been tied to many other physiological functions, including motivation and reward (Fragale et al., 2021b). For example, evidence from the clinical setting shows that narcoleptic patients seldom abuse their medication, which in many cases consists of amphetamine-based stimulants with known addictive potential (Turner, 2019). In addition, postmortem studies have shown a marked increase in orexin-immunoreactive neurons in the brains of opiate users compared to non-users (Thannickal et al., 2018), an observation further verified in animal models of the self-administration of fentanyl and cocaine (Fragale et al., 2021a; James et al., 2019) and in embryonic exposure to ethanol (Collier et al., 2022). Together, this suggests the involvement of orexin activity in the development of SUDs. In this regard, studies carried out in animal models have shown that the number of orexinergic cells in the lateral hypothalamus is a predictor of addiction vulnerability, as individuals with higher cell counts in this region demonstrate increased motivation for cocaine consumption (Pantazis et al., 2020). The observed upregulation of orexinergic cells associated with substance use hints towards the existence of a plastic mechanism that could help explain the development of SUDs (and possibly insomnia, as both behaviors have been shown to relate to augmented orexinergic stimuli). While neurogenesis could explain the increase in orexin-expressing cells, evidence suggests this is not the case, as markers for new cells, such as bromodeoxyuridine (BrdU) or doublecortin, do not appear increased under conditions of drug administration (Thannickal et al., 2018). These observations have led researchers to suggest the existence of a reserve population of orexinergic cells among which peptide production is transiently induced in response to environmental demands but can become sustained as a result of chronic activation, such as after long-term exposure to substance abuse (James & Aston-Jones, 2022).

Further evidence in favor of the role of orexinergic stimulation in addictive behaviors has accumulated over the years. For example, the mapping of orexinergic projections has shown that many limbic structures, such as the nucleus accumbens and ventral tegmental area, receive orexinergic input from the lateral hypothalamus (Peyron et al., 1998). This connectivity is functional, as it has been shown that orexin peptides administered into the ventral tegmental area can elicit dopamine release into the nucleus accumbens and prefrontal cortex (Kalló et al., 2022), two sites, respectively, described as relevant for the binge/intoxication and preoccupation/anticipation stages of the addiction cycle. In addition, orexin activity appears to be involved in the establishment and maintenance of drug-seeking behavior, as preclinical studies show that the administration of orexinergic antagonists facilitates extinction and reduces the self-administration of different substances such as methamphetamine (Zamanirad et al., 2023), alcohol (Flores-Ramirez et al., 2022), and cannabis (Flores et al., 2014), to cite some. The positive results obtained from animal models have allowed for the study of orexinergic antagonists as therapeutic targets for the treatment of SUDs, with suvorexant and lemborexant currently being tested in clinical trials for the treatment of alcohol, cocaine, nicotine, opioid, and methamphetamine use disorders. In this regard, suvorexant has already yielded positive results as it has been shown to be able to improve sleep disturbances and reduce cravings in patients recovering from opioid use disorder (Huhn et al., 2022), which is clear evidence that orexin antagonists, such as suvorexant, are promising candidates for the treatment of SUDs (James et al., 2020).

In sum, the increasing amount of evidence has led some authors to argue that orexin neurotransmission represents a biological link between sleep–wake regulation and addiction. For example, Fragale et al. maintain that increased orexinergic tone mediates an insomnia–addiction loop, where increased orexin tone compromises sleep, causing impaired cognitive function and increasing substance use, which further boosts orexinergic signaling and the worsening of insomnia (Fragale et al., 2021b).

## 6. Conclusions

**Figure 1:**
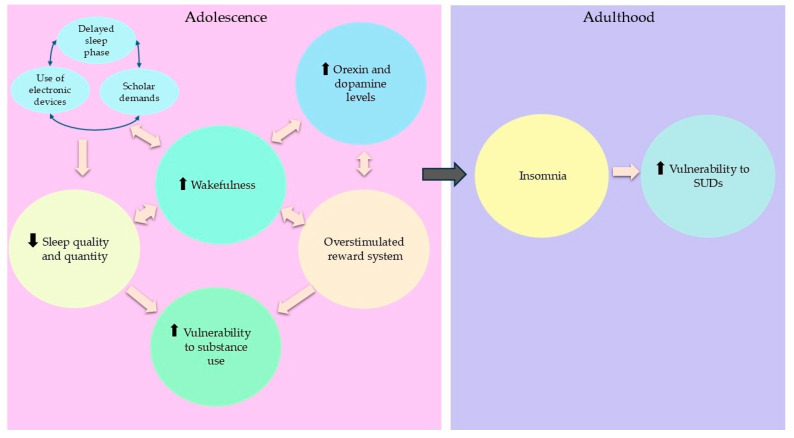
Various factors, such as a delayed sleep phase, social jet lag, school schedules, and the use of electronic devices, alter sleep habits during adolescence, reducing sleep duration and quality. This, in turn, leads to increased periods of wakefulness, which elevates the release of orexin and dopamine in the brain, overstimulating the reward system and making adolescents more vulnerable to substance use. If these factors are perpetuated in an adolescent, insomnia appears during adulthood and SUD vulnerability increases.

During adolescence, sleep plays a critical role in supporting key processes of maturation and learning. However, a combination of biological factors, such as a delayed sleep phase and social aspects, including school schedules, the frequent use of electronic devices, and social jet lag, often reduce sleep duration and quality (Figure 1). In this regard, available studies coincide in that adolescents tend to sleep less than the recommended amount, at an average of 8.5 h per night at the age of 13. Lack of sleep is then maintained until late adolescence, with sleep duration declining by approximately another 1.5 h by the age of 18 (Maslowsky & Ozer, 2014). Moreover, the underdeveloped prefrontal cortex, associated with the heightened impulsiveness characteristic of adolescence, contributes to increased risk-taking behaviors, including substance use. This impulsiveness, in addition to recurrent sleep issues, makes adolescents particularly vulnerable to the development of SUDs, as evidenced by studies that indicate that a significant proportion of substance users initiate consumption at 14 years old with a consequent increased risk of becoming poly-users later on (Dokkedal-Silva et al., 2021; Hasler et al., 2022; Haynie et al., 2018; Leger et al., 2022; Liu et al., 2023; Miller et al., 2017; Nguyen-Louie et al., 2018; Zahran & Janssen, 2023).

On the other hand, it has been vastly documented that the orexinergic system plays an important role in both the maintenance of arousal and in motivation and reward, allowing this system to be proposed as the underlying link between the high frequency of co-occurring sleep disorders and SUDs (Gyawali & James, 2023; Mohammadkhani et al., 2024). Additional evidence suggests that the activity of this system is increased in conditions of extended vigilance and through addiction (Bjorness & Greene, 2024).

This, for example, has been noted in the case of adult insomnia patients (Tang et al., 2017) and adolescents diagnosed with internet gaming disorder (Choi et al., 2020), two groups that have been shown to possess increased orexin plasma levels. These findings further support the notion that such conditions occur with exacerbated orexinergic activity, as they contrast with what has been observed in the general population, where orexin levels tend to remain similar regardless of age and gender (Kanbayashi et al., 2002). In addition, evidence obtained from animal models provides further clues in support of the orexinergic role in sleep and addiction. For instance, it has been documented that orexinergic activity is highest during wake and sleep restriction (Estabrooke et al., 2001; Modirrousta et al., 2005) and that rats repeatedly exposed during adolescence to binge-levels of ethanol demonstrated an increase in the number of orexin-expressing cells when evaluated in adulthood (Amodeo et al., 2020). Thus, we hypothesize that chronic insufficient sleep during adolescence could lead to maladaptive changes in the orexinergic system, causing increases in orexin activity and the number of orexin-expressing cells that facilitate the development of SUDs and insomnia further in life (Figure 1).

All this highlights the importance of observing adolescent sleep patterns and studying substance use during this stage, as the bidirectional interactions between substance use and sleep disruption contribute to a detrimental cycle that exacerbates the many challenges faced during adolescence and cause detrimental effects that can continue into adulthood.
